# The Effects of COVID-19 Lockdown on Health and Psychosocial Functioning in Older Adults Aged 70 and Over

**DOI:** 10.1177/23337214211039974

**Published:** 2021-09-06

**Authors:** Sarah Docherty, Crystal F. Haskell-Ramsay, Lynn McInnes, Mark A. Wetherell

**Affiliations:** 1 Department of Psychology, 5995 Northumbria University, Newcastle upon Tyne, UK

**Keywords:** lockdown, COVID-19, older adults, psychosocial well-being, physical health, physical activity

## Abstract

The COVID-19 pandemic led to a state-imposed lockdown in the UK; there are many psychosocial consequences of pandemics, with older adults potentially at an increased risk. The current study assessed psychosocial functioning in a sample of older adults in the UK with baseline data collected pre-lockdown and follow-up 12 weeks later during lockdown. Thus, allowing investigation of the effect of COVID-19 and associated lockdown on psychosocial well-being. Thirty-five older adults (Mean age = 76.06, sex = 12 males) participated in this repeated measures study. A final follow-up was then conducted post-lockdown to capture any factors that were viewed as helpful to well-being during lockdown. From pre- to during lockdown, perceived stress, well-being, depressive symptoms, mood disturbance and memory were all significantly worsened. There were significant improvements in self-reported physical health symptoms, social interaction, time spent engaging in physical activity and certain aspects of relationship quality. Follow-up showed that well-being, depression and mood were still negatively affected post-lockdown. Given the sample were all ‘healthy’ at baseline in comparison to established norms, there may be greater impairment in more vulnerable populations. Support for older populations is needed to mitigate the negative effects shown, particularly in light of the endurance of some of these effects post-lockdown.

## Introduction

The World Health Organization (WHO) declared COVID-19 a global pandemic in March 2020; there have been in excess of 92 million confirmed cases worldwide (World Health Organization, 2020a), and in the UK, in excess of 3.2 million cases and more than 90,000 deaths (UK, GOV 2020). International responses were implemented to contain the spread of the virus, with various states of imposed lockdown applied in most European countries (Brodeur et al., 2020).

There are many psychosocial consequences of pandemics, with research showing that individuals’ mental health has been severely affected by COVID-19 and the associated lockdown (Brodeur et al., 2020). In a sample of 775 adults in the United States, 55% reported that COVID-19 had negative effects on their mental well-being (World Health Organization, 2020b). Research has also shown higher rates of mental distress during lockdown (Sibley et al., 2020) and it has been hypothesised that frustration, boredom, low mood and potentially depression are likely consequences (Venkatesh & Edirappuli, 2020). Some groups are particularly vulnerable including those who contract the disease, those at a heightened risk of contraction and people with pre-existing medical, psychiatric or substance use problems (Pfefferbaum & North, 2020). The older adult population are at a heightened risk for contracting COVID-19 and often have pre-existing medical conditions which places them at increased risk of these additional consequences.

Older adults have been directly impacted by many of the implemented policies to mitigate the pandemic, including self-isolation procedures. As the older population often rely on community centres, social groups and places of worship for social interaction (Armitage & Nellums, 2020), the shielding policies may have disproportionally affected them. This suggests that they may therefore require more effective psychosocial support during this time (Kuwahara et al., 2020), with the WHO highlighting older adults may have a higher chance of becoming anxious, angry, stressed, agitated and withdrawn during the outbreak or while in quarantine (World Health Organization, 2020b).

There has been a wealth of research assessing well-being; however, as the pandemic and resulting lockdown were unanticipated, few studies have data relating to before the lockdown. Pre-lockdown data would provide a baseline comparison and would more effectively allow for the assessment of how the current pandemic and resulting restrictions have affected mental health and well-being (Brodeur et al., 2020). The current study can address this issue. As part of a longitudinal study assessing well-being and everyday functioning in older adults, data were collected in a population of over 70s prior to the UK lockdown in March 2020. For participants in this study, one scheduled follow-up session coincided with the lockdown period. Comparison of the baseline and follow-up therefore allowed for the direct effects of COVID-19 lockdown on psychosocial well-being to be studied in a population who are at increased risk of being negatively impacted by these restrictions**.**

## Methods

### Design

The current study utilised a quantitative repeated measures design. The repeated factor was time which had two levels; baseline (pre-lockdown) and follow-up (during lockdown), which were separated by 12 weeks. Pre-lockdown data was collected Jan–March 2020 and during lockdown data March–June 2020. Additionally, participants were invited to complete a third time point (post-lockdown collected in May 2021) in order to follow-up on any lasting impact. The dependent variables assessed the following dimensions: well-being, stress, general health, daily functioning, mood trait measures, sleep quality, memory, activity levels, fear of falling, social network size and loneliness. Participants were originally enrolled into a randomised, placebo-controlled, double-blind, independent groups study, assessing the effects of a multi-nutrient supplement on everyday functioning in older adults (Clinical Trials ID NCT04112732).

### Participants

The sample consisted of 35 participants who completed a baseline assessment pre-lockdown and a follow-up during lockdown, average age 76.06 (SD = 4.60), with ages ranging between 70 and 90. This consisted of 12 men (mean age =75.58, SD = 4.06) and 23 women (mean age = 76.30, SD = 4.93). The average BMI in the sample was 26.71 kg/m^2^, participants on average reported consuming 1.22 units of alcohol and 305.2 mg of caffeine per day. All participants were from a white ethnicity and had on average 15.1 years education (SD = 3.44). No participants had any food allergies, epilepsy, haemochromatosis or were under medical supervision, and all were non-smokers. Four participants had a thyroid disorder and consulted their doctor/pharmacist before taking part. No participants were currently taking multi-nutrient supplements; consumption of other supplements (e.g. turmeric, cod liver oil, glucosamine and rose hip) was considered on a case-by-case basis. Participants were reimbursed either £50 or £65 for their time (depending on what aspects of the original intervention study they signed up to).

Twenty-three participants completed post-lockdown measures, average age 75.65 (SD = 3.84). This consisted of nine men (mean age = 75.44, SD = 3.94) and 14 women (mean age = 75.79, SD = 3.93).

### Materials

Full descriptions of questionnaire materials including scoring can be found in Clinical Trials registration (ID NCT04112732).

#### Well-being

UK Office of National Statistics (ONS) four subjective well-being questions (ONS4) (Tinkler, 2015). An additional question was included which was: Overall, how well did you feel yesterday?

#### Stress

The Perceived Stress Scale (PSS) (Cohen et al., 1983) measures the extent to which participants perceive their lives to be overwhelming, uncontrollable and unpredictable.

#### General health

The Cohen–Hoberman Inventory of Physical Symptoms (CHIPS) (Cohen & Hoberman, 1983) which consists of 33 common symptoms (e.g. ‘back pain’ and ‘constipation’).

The SF-20 measured general health across six domains: physical functioning, role functioning, social functioning, mental health, health perceptions and pain (Stewart et al., 1988).

#### Daily functioning

The Instrumental Activities of Daily Living (IADL) (Lawton & Brody, 1969) was used to measure how an individual is functioning at the present time. This measures eight daily activities: telephoning, shopping, food preparation, housekeeping, laundering, use of transportation, use of medicine and financial behaviour.

#### Mood trait and state measures

The Hospital Anxiety and Depression Scale (Zigmond & Snaith, 1983) was used to measure depression and anxiety, which can indicate borderline and probable mood disorders (Snaith, 2003).

The Profile of Mood States (McNair et al., 1971) comprises of 65 adjectives (e.g. helpful, unhappy) which gives six global scores: tension, depression, anger, fatigue, confusion and vigour and one total mood disturbance score.

#### Sleep quality

The Pittsburgh Sleep Quality Inventory (PSQI) (Buysse et al., 1989) was used to measure sleep quality and patterns. This assesses seven domains (subjective sleep quality, sleep latency, sleep duration, habitual sleep efficiency, sleep disturbances, use of sleep medication and daytime dysfunction) and one global sleep score.

#### Memory

The Prospective and Retrospective Memory Questionnaire (PRMQ) (Crawford et al., 2003) measured everyday memory. This measures memory failures in two subscales, prospective memory failures and retrospective failures.

#### Activity levels

The Yale Physical Activity Survey (Dipietro et al., 1993) was used to assess physical activity levels. This gives indications of weekly energy expenditure, total time index and overall activity dimension summary index.

#### Fear of falling

Concerns about falling were measured using the Falls Efficacy Scale International (Yardley et al., 2003 2005). This includes 16 items, and participants were asked to rate how much they would be concerned with falling while doing this activity.

#### Social network size

The Convoy Method (Antonucci & Akiyama, 1987) measured social network size, which measures number of individuals in different social networks, quality of these relationships and total social network size.

The Lubben Social Network Scale (Lubben, 1988) was also used to measure social networks and social engagement. Questions relate to different aspects of social networks such as active social network, perceived support network and perceived confidant network.

#### Loneliness

Loneliness was measured using the 11-item De Jong Gierveld Loneliness Scale (De Jong-Gierveld & Kamphuls, 1985), which measures emotional loneliness and social loneliness. A single index of loneliness can also be produced by totalling these two scores where higher scores indicate greater loneliness.

### Procedure

Participants completed the initial testing visit during a face-to-face session in the laboratory. Participants had been asked to avoid caffeinated products for 12 hours and alcohol for 24 hours. They were instructed to eat a breakfast of cereal and/or toast at least 1 hour before the visit began. On arrival, participants gave written informed consent, provided lifestyle and demographic data and completed paper questionnaires. This took around 1 hour in total. Additional tasks of mobility, strength, cognitive demand and stress reactivity were then completed, and participants were given treatment (either multi-nutrient or placebo) to take for the following 12 weeks. These tasks and activities are not reported here as they were not completed in the follow-up session (full details can be found at clinicaltrials.gov ID NCT04112732).

All participants completed visit one before lockdown restrictions were put in place due to COVID-19. As all face-to-face research was prohibited, baseline questionnaires were amended for online completion at follow-up during lockdown.

Participants completed their follow-up visit from home, 12 weeks (+/− 5 days) after their baseline assessment. For consistency, participants were asked to adhere to the same instructions outlined for the baseline visit. The online questionnaire link was sent via email and participants worked through the questionnaires at their own pace. After completing the questionnaires, participants were debriefed and directed to a portal for participant payment.

In May 2021, participants were contacted and asked to complete the same questionnaires again adhering to the same instructions as outlined above. Additionally, participants were asked nine open-ended qualitative questions regarding what they found helpful to coping and reducing any negative effects of lockdown. These questions were relating to: socialising, community support, digital support and lifestyle.

### Treatment of Data

All data was analysed using IBM SPSS statistics version 26. Descriptive statistics were calculated for all measures. Outcome measures were analysed using repeated measures ANOVA; time was the within-subjects factor which consisted of two levels, pre-lockdown and during lockdown. Separate repeated measures ANOVA were then conducted on pre-lockdown and post-lockdown outcome measures. The dependent measure was the relevant outcome for each questionnaire. All descriptive statistics, F and *p* values for all outcome measures are displayed in Tables 1–3, only significant analyses and effect sizes are reported in text.

**Table 1.** Mean (SD), sample size (N) for all well-being, mood and memory outcome measures pre-lockdown and during lockdown.

	N	Pre-Lockdown	During Lockdown	F Value	*p* Value
Perceived Stress Scale	32	11.09 (6.08)	13.13 (8.03)	5.57	.025
Office National Statistics well-being	33	40.00 (7.75)	37.39 (7.78)	4.95	.033
Hospital Anxiety and Depression Scale					
Anxiety	33	4.55 (3.46)	5.00 (4.05)	1.00	.325
Depression	33	2.64 (2.66)	3.58 (3.57)	7.18	.012
Profile of mood states					
Tension–anxiety	30	3.97 (5.07)	6.17 (7.85)	3.84	.060
Depression–dejection	30	2.53 (7.90)	5.10 (8.97)	11.76	.002
Anger–hostility	30	1.90 (6.81)	3.5 (5.32)	2.06	.162
Vigour–activity	30	22.70 (6.81)	21.20 (8.07)	1.44	.240
Fatigue–inertia	30	1.97 (3.93)	2.90 (5.52)	2.20	.148
Confusion–bewilderment	30	4.73 (5.47)	6.47 (6.28)	8.49	.007
Friendliness	30	18.37 (3.74)	17.63 (4.84)	0.81	.376
Total mood disturbance	30	−7.60 (30.38)	2.93 (36.37)	7.94	.009
Prospective and Retrospective Memory Questionnaire					
Prospective memory	32	21.22 (6.85)	29.78 (5.67)	34.15	<.001
Retrospective memory	32	20.53 (7.68)	30.19 (6.37)	28.31	<.001

**Table 2.** Mean (SD), sample size (N) for all physical health and activity measures pre-lockdown and during lockdown.

	N	Pre-Lockdown	During Lockdown	F Value	*p* Value
Cohen–Hoberman Inventory of Physical Symptoms	32	14.25 (12.65)	10.38 (12.99)	6.67	.015
Yale Physical Activity Scale					
Total time	31	31.58 (21.47)	44.70 (24.33)	5.63	.024
Energy expenditure	31	115.58 (101.31)	149.43(79.63)	2.74	.108
Activity dimension	31	58.13 (29.12)	55.74 (26.55)	0.15	.699
Pittsburgh Sleep Quality Index	32	8.09 (3.52)	7.50 (3.57)	1.45	.238
Instrumental activities of daily living	32	7.47 (1.14)	7.47 (1.16)	.00	1
Falling Efficacy Scale- International	32	21.28 (8.18)	20.84 (8.18)	.21	.644
SF-20					
Physical function	32	74.22 (28.91)	69.76 (28.64)	1.34	.254
Role functioning	32	80.23 (36.42)	83.72 (37.35)	0.69	.412
Social functioning	32	90.70 (15.34)	86.98 (29.88)	0.86	.358
Mental health	32	85.02 (15.81)	81.95 (16.78)	2.49	.122
Health perceptions	32	75.30 (20.38)	78.02 (22.36)	1.16	.288
Pain	32	36.74 (27.23)	33.85 (29.77)	0.72	.403

**Table 3.** Mean (SD), sample size (N) for all social interaction and loneliness measures pre-lockdown and during lockdown.

	N	Pre-Lockdown	During Lockdown	F Value	*p* Value
Lubben Social Network Scale	32	33.91 (10.42)	36.00 (9.96)	4.46	.043
Convoy model social relations					
Inner circle relationships	32	7.26 (5.07)	6.84 (4.06)	0.24	.630
Inner relationship quality	32	8.43 (2.39)	8.48 (1.82)	0.07	.796
Middle circle relationships	32	5.69 (3.52)	4.38 (2.78)	1.32	.260
Middle relationship quality	32	6.45 (2.97)	6.75 (2.31)	0.50	.483
Outer circle relationships	32	4.71 (5.97)	3.84 (2.27)	0.27	.611
Outer relationship quality	32	4.50 (3.39)	6.26 (2.47)	8.61	.006
Total relationships	32	17.66 (10.38)	15.06 (7.24)	0.72	.402
Total relationship quality	32	7.43 (2.18)	7.38 (1.64)	0.00	.990
DeJong Loneliness Scale					
Social loneliness	32	1.66 (1.96)	1.66 (1.45)	.00	1
Emotional loneliness	32	1.53 (2.12)	1.47 (1.95)	.04	.845
Total loneliness	32	3.19 (3.77)	3.13 (2.74)	.02	.902

Qualitative data was analysed per question, responses were read by the lead researcher and recurring ideas were coded and grouped together to form overarching ideas, which were then combined due to overlap in responses. This analysis was supplementary to the main results to suggest protective factors against isolation and will be highlighted in the discussion but not reported in the results section.

## Results

### Well-being, Mood & Memory

Participants reported significantly lower levels of well-being [F (1, 32) = 4.95, *p* = .033, d = 0.34], greater levels of perceived stress [F (1, 31) = 5.57, *p* = .025, d = 0.29] and depressive symptoms [F (1, 32) = 7.18, *p* = .012, d = 0.3) and higher scores for the mood states of depression–dejection [F (1, 29) = 11.76, *p* = .002, d = 0.3]; confusion–bewilderment [F (1, 29) = 8.49, *p* = .007, d = 0.3] and total mood disturbance [F (1, 29) = 7.94, *p* = .009. d = 0.31) during lockdown compared with pre-lockdown, and there was a trend towards greater levels of tension/anxiety [F (1, 29) = 3.84, *p* =.06, d = 0.33]. Participants reported significantly more memory failures during lockdown for both prospective [F (1, 31) = 34.15, *p* <.001, d = 1.36] and retrospective [F (1, 31) = 28.31, *p* <.001, d = 1.37] memory compared to pre-lockdown. All outcomes are shown in Table 1.

### Physical Health and Activity

There was a significant reduction in CHIPS scores, indicating improved physical health during lockdown [*F* (1, 31) = 6.67, *p* = .015, d = 0.3]. There was a significant increase in time spent engaging in physical activity during lockdown compared to pre-lockdown [*F* (1, 30) = 5.63, *p* =.024, d = 0.57]. All outcomes are shown in Table 2.

### Social Interaction and Loneliness

Social interaction as measured by the Lubben Social Networks Scale significantly increased from pre-lockdown to during lockdown [F (1,31) = 4.46, *p* = .043. d = 0.21]. In terms of social network dynamics, participants reported greater levels of relationship quality with those in their outer circle during lockdown compared to pre-lockdown [F (1, 31) = 8.61, *p* = .006, d = 0.59]. All outcomes are shown in Table 3.

### Follow-up

Additional ANOVAs were conducted on the subsample between baseline and post-lockdown to assess whether any changes remained after lockdown ended.

Of the outcomes significantly affected; well-being [F (1, 22) = 10.81, *p* =.003], HADs depression [F (1, 22) = −7.64, *p* = .011] and POMS total mood disturbance [F (1, 22) = 4.72, *p* = .041] continued to be negatively affected post-lockdown in this sub-sample.

POMS depression [F (1, 22) = 2.25, *p* = .148], POMS confusion [F (1, 22) = .90, *p* = .354], retrospective memory [F (1, 22) = 2.99, *p* = .098] and prospective memory [F (1, 22) = 2.98, *p* = .098] were no longer impacted post-lockdown.

No significant effects were observed on PSS, physical activity, CHIPS, social networks or loneliness in this sub-sample.

## Discussion

The current study assessed the effects of the nationwide COVID-19 lockdown on a range of measures of well-being in over 70s in the UK. Importantly, pre-lockdown data were available, which allowed the direct effects to be studied prospectively in a population who are likely to have been significantly impacted by the restrictions imposed. Results showed that there were largely negative implications for well-being, mood, perceived stress and memory, although some improvements were shown in general health, physical activity and social interaction.

Firstly, lockdown led to significantly decreased feelings of well-being, increased feelings of depression and confusion, greater total mood disturbance and a trend towards greater feelings of tension and anxiety. This is consistent with previous research in New Zealand showing that lockdown can lead to higher levels of mental distress, low mood and depression when compared to a matched sample (Sibley et al., 2020; Venkatesh & Edirappuli, 2020). The current results strengthen this conclusion by replicating findings in participants measured pre-and during lockdown rather than through comparisons with a matched sample.

The observed deterioration in mood could be due to the significant increases in perceived levels of stress. Research from previous crises, such as the severe acute respiratory syndrome pandemic and Ebola virus, have shown that such situations increase stress levels and have negative mental health implications (Mak et al., 2009; Cénat et al., 2020). Given the scale and severity of the current situation, it is not surprising that stress levels significantly increased, and this highlights the importance of identifying ways to minimise potential negative consequences. The increased levels of stress may also provide an explanation for the detrimental effects on memory, as greater recent life stress has been associated with more self-reported memory problems (Shields et al., 2017). This is the first study to highlight that there may be detrimental cognitive consequences of lockdown in older adults. This is particularly important as stressful events in older adults can trigger a cognitive decline, with many reporting a stressful event before the onset of dementia (Tsolaki et al., 2010). The early identification of memory problems could therefore mitigate against longer term consequences.

It is noteworthy that pre-lockdown scores for stress, anxiety and depression fall below the norms for these measures (Cohen et al., 1983; Crawford et al., 2001) indicating a relatively healthy sample; however, scores exceed norm values during lockdown. If such deteriorations are observed in relatively healthy participants, the impact in populations who may already show abnormal/clinical symptoms is of greater concern.

In contrast, there was a significant improvement in self-reported physical health during lockdown. There are a number of possible explanations for this. It is plausible that those in this age group, who are likely to be vulnerable, may be making a more concerted effort to improve their health status. Alternatively, as most facilities were closed during the lockdown period, it is possible participants were alleviated from many of their normal day-to-day duties and therefore had more time to rest, meaning physical symptoms such as muscular pain were reduced. It could also be suggested the benchmark for perceiving physical health has increased due to relief of not experiencing COVID symptoms or not wanting to present any signs of illness.

There was a significant increase in the total time engaging in physical work, exercise and recreational activities during lockdown. This may seem counterintuitive given that most, if not all, recreational activities would have been suspended during this time. However, data analysing Google trends showed an increase in interest in exercise immediately after lockdown in the UK (Ding et al., 2020). Potential explanations included compensation for reduced incidental activities, increased expendable time, more awareness of one’s own health and lockdown rules explicitly allowing for exercise as an essential activity. Due to the nature of the measure of physical activity used in this study, this could also reflect an increase in physical activity in the home during this time, as the range of activities listed includes housework and gardening activities. Lockdown coincided with the sunniest spring on record in the UK (Taylor, 2020), which could give the opportunity for more outdoor physical activity. Given that most of the individuals in this sample would have been instructed to stay at home during this time, it seems a plausible explanation that they may have increased the amount of activity completed at home.

Two aspects of social interaction were also improved during lockdown, indicating that lockdown may have had beneficial social implications in this population. Firstly, the Lubben Social Network Scale indicated that participants had increased social engagement. Secondly, there was a significantly improved rating of relationship quality in the outer circle of The Convoy Method of social relationships. This circle is for people who may be at the peripheral of an individual’s social network but are close enough and important enough to be part of their personal network. These results seem contradictory to much of the published literature in this area which would predict elevated levels of loneliness and social isolation in this population due to social distancing measures (Hwang et al., 2020). However, the current crisis has led to an increase in community spirit, with online interaction increasing 82% within the first month of lockdown, mainly concerning support for the most vulnerable, particularly the elderly (Weston, 2020). This could explain improvements in relationship quality with those in extended aspects of social networks; contact with these individuals appears to have increased due to the re-emphasis on community spirit. It is interesting that much published work anticipated that it would be social isolation which led to worsened mental and physical health (Armitage & Nellums, 2020; Webb, 2020). However, this is not evident in the current study, which observed improvements in reports of social interaction and no changes in levels of loneliness.

There is an urgency to study the mental health impact of COVID-19 in real time so that the adverse impact can be anticipated and minimised (Vahia et al., 2020). These findings address this need and help to understand the impact of the pandemic on mental health and well-being which will prepare for future pandemics, as well as ongoing national and local lockdowns and identify where support is needed. Our follow-up data indicated that well-being and aspects of mood (depression and mood disturbance) were still negatively affected post-lockdown suggesting enduring effects of the pandemic and associated lockdown.

It is important to identify potential protective factors to the detrimental consequences observed and to explore any preventative behaviours the older population can adopt to protect themselves against a chronic stressor such as a pandemic or to help with isolation in general. Participants discussed how keeping in contact with friends and family via Zoom/FaceTime helped throughout, but for some individuals, there was a need for support/guidance on the practical and technical aspects of how to do this. This highlights the need for technological assistance in older adults which may help combat feelings of loneliness and isolation. Additionally, participants stated how being part of wider groups helped feelings of isolation such as online worship and Zoom exercise classes; this may be a helpful alternative for older adults who cannot travel/attend in person activities going forward. In terms of lifestyle factors, participants indicated that regular exercise, in particular going out for walks, was beneficial throughout lockdown. These findings suggest this should be encouraged in older adults especially those who live alone. Although these questions were discussed in respect to the pandemic, they have useful implications for tackling isolation and loneliness in older adults in general.

## Conclusion

Overall, the findings from the current study provide evidence of both negative and positive consequences of lockdown. The impact of the COVID-19 pandemic lockdown in a sample aged 70 and over in the UK is therefore mixed. Unlike other studies that have attempted to assess the impact of lockdown, this is the first study to research this population in the UK with initial measurement collected before lockdown. Negative impacts were observed despite improvements to physical health and increases in physical activity and social interaction. Given the sample were all ‘healthy’ at baseline in comparison to established norms, there may be greater impairment in populations who are unable to increase their activity, are more socially isolated or already show clinical mood symptoms pre-lockdown.
